# Enhancing the Performance and Stability of Li-CO_2_ Batteries Through LAGTP Solid Electrolyte and MWCNT/Ru Cathode Integration

**DOI:** 10.3390/nano14231894

**Published:** 2024-11-26

**Authors:** Dan Na, Dohyeon Yu, Hwan Kim, Baeksang Yoon, David D. Lee, Inseok Seo

**Affiliations:** 1 Department of Electronic and Information Materials Engineering, Division of Advanced Materials Engineering, Research Center of Advanced Materials Development, Jeonbuk National University, Jeonju 54896, Republic of Korea; ld3310@jbnu.ac.kr (D.N.); yudohyeon@jbnu.ac.kr (D.Y.); rlaghks0202@jbnu.ac.kr (H.K.); qortkd2134@jbnu.ac.kr (B.Y.); 2 Aerospace Engineering, Iowa State University, Ames, IA 50011, USA; daylee@iastate.edu

**Keywords:** Li-CO_2_ battery, solid electrolyte, NASICON, Ru catalyst

## Abstract

Li-CO_2_ batteries (LCBs) have emerged as promising solutions for energy storage, with the added benefit of contributing to carbon neutrality by capturing and utilizing CO_2_ during operation. In this study, a high-performance LCB was developed using a Ge-doped LiAlGeTi (PO_4_)_3_ (LAGTP) solid electrolyte, which was synthesized via a solution-based method by doping Ge into NASICON-type LATP. The ionic conductivity of the LAGTP pellets was measured as 1.04 × 10^−3^ S/cm at 25 °C. The LCB utilizing LAGTP and an MWCNT/Ru cathode maintained a stable cycling performance over 200 cycles at a current density of 100 mA/g, with a cut-off capacity of 500 mAh/g. Post-cycle analysis confirmed the reversible electrochemical reactions at the cathode. The integration of LAGTP as a solid electrolyte effectively enhanced the ionic conductivity and improved the cycle life and performance of the LCB. This study highlights the potential of Ge-doped NASICON-type solid electrolytes for advanced energy-storage technologies and offers a pathway for developing sustainable and high-performance LCBs.

## 1. Introduction

With growing concerns regarding global warming and environmental deterioration, the reduction of CO_2_ emissions has become an urgent global priority. As a greenhouse gas, CO_2_ contributes significantly to climate change, highlighting the need for innovative strategies for its reduction and utilization [1,2]. Li-CO_2_ batteries (LCBs) have emerged as a promising solution, offering the dual advantages of high energy density and CO_2_ sequestration [3,4]. LCBs operate through reversible electrochemical reactions that enable simultaneous energy storage and carbon capture [5,6].

(1) $$4{\mathrm{Li}}^{+}+4e^{-}+3{\mathrm{CO}}_{2}\;\to 2{\mathrm{Li}}_{2}{\mathrm{CO}}_{3}+C$$

This reaction provides a high theoretical energy density of up to 1876 Wh/kg, making LCBs attractive for applications in CO_2_-rich environments and contributing to efforts toward achieving carbon neutrality [7]. However, conventional LCBs typically employ organic liquid electrolytes, which present several challenges such as flammability, volatility, leakage risks, and inadequate suppression of lithium dendrite formation [8,9]. These issues compromise the safety and hinder the practical application of LCBs. Solid-state electrolytes have been introduced to address these limitations, offering enhanced safety and electrochemical performance in terms of safety and stability [10,11].

Lithium aluminum titanium phosphate (LiAlTi (PO_4_)_3_, LATP), which has a sodium superionic conductor (NASICON) structure, is a well-known solid electrolyte characterized by high ionic conductivity and excellent chemical and thermal stabilities [12]. The performance of LATP can be further enhanced by doping it with various metals. Doping expands the Li-ion migration pathways and optimizes the lattice structure, leading to increased ionic conductivity and an expanded electrochemical-stability window, which facilitates stable operation at higher voltages [13,14]. Building on these advantages, Ge doping has been explored to improve the properties of LATP further. By substituting Ti^4+^ with Ge^4+^ ions, lithium-aluminum-germanium-titanium phosphate (LiAlGeTi (PO_4_)_3_, LAGTP) can be formed from LATP. Ge doping induces lattice distortions that adjust lattice constants and improve ionic conductivity by reducing the migration energy barrier. This process facilitates lithium-ion migration by creating interconnected diffusion pathways [15].

In addition to optimizing the electrolyte, developing efficient cathode materials is essential for enhancing LCB performance. Multi-walled carbon nanotubes (MWCNTs) are widely used as cathodes because of their high electrical conductivity and large surface area; however, they have limited catalytic activity for the decomposition of Li_2_CO_3_ [16]. The catalytic activity of MWCNTs can be significantly enhanced by introducing an Ru catalyst, effectively reducing overpotentials and improving battery efficiency and cycle life. The Ru catalyst lowers the activation energy required for the decomposition of Li_2_CO_3_, reducing the polarization and further improving battery performance [17].

In this paper, we present a high-performance LCB that integrates LAGTP solid electrolytes with Ru-catalyzed MWCNT cathodes. Ge doping enhances the electrolyte’s ionic conductivity and structural stability, and the Ru catalyst improves electrochemical reactions involving CO_2_. We evaluated the electrochemical properties of the fabricated LCB, and the results demonstrated significant performance enhancements due to the synergistic effects of the optimized solid electrolyte and the MWCNT/Ru cathode material. This approach offers a promising pathway for developing safe, efficient, and high-energy-density LCBs for advanced energy storage applications.

## 2. Materials and Methods

### 2.1. Preparation of LAGTP Pellet

LAGTP powder was synthesized using a solution-based method. Initially, LiCl (Samchun, Seoul, Republic of Korea, 98.2%), NH_4_H_2_PO_4_ (Samchun, 98.0%), and Al (NO_3_)_3_·9H_2_O (Samchun, 98.0%) were dissolved stoichiometrically in deionized water with magnetic stirring. Subsequently, GeO_2_ (99.9%, Sigma-Aldrich, St. Louis, MI, USA) was added to the mixture and pulverized in a high-energy mill (Taemyong Scientific, Uiwang-si, Republic of Korea). Titanium butoxide (Samchun, 97.0%) was added to the solution, followed by continuous magnetic stirring. The resulting precipitates were homogeneously mixed using a planetary mill (Pulverisette 5, Fritsch, Idar-Oberstein, Germany). After drying in an oven at 80 °C for 24 h, the mixture was calcined at 800 °C for 10 h to eliminate volatile impurities. Polyvinyl alcohol (3wt%) was then added to the mixture and pressed into pellets using a tungsten carbide die at 60 MPa. The pellets were sintered at 950 °C for 12 h and polished to achieve the desired thickness and surface smoothness for cell applications.

### 2.2. Preparation of the Cathode (MWCNT and MWCNT/Ru Powder)

RuCl_3_·xH_2_O (Sigma Aldrich, 50 mg) was added to ethylene glycol (Daejung, Goryeong-gun, Republic of Korea, 99.0%, 150 mL) and dissolved through magnetic stirring. Subsequently, MWCNT (Sigma Aldrich, 60 mg) was introduced into the solution and sonicated for 1 h. The mixture was then refluxed at 180 °C for 3 h. After cooling to room temperature, the supernatant was decanted, and the mixture was centrifuged to separate the MWCNT/Ru from solution, which were then rinsed with ethanol and deionized water. The collected solids were dried in a vacuum oven at 70 °C for 12 h. To prepare the cathode material, MWCNT and MWCNT/Ru powders (80 wt%) were separately blended with polyvinylidene fluoride (PVDF, 20 wt%) and dispersed in N-methyl-2-pyrrolidone (NMP) using a non-bubbling kneader. The resultant paste was uniformly applied onto a carbon cloth substrate with 14 mm diameter and dried in a vacuum oven at 70 °C.

### 2.3. Li-CO_2_ Cell Assembly Procedure

For the electrochemical evaluation of the proposed LCB, we used a CR-2032 coin cell that featured a hole on its side for CO_2_ gas exposure. The LCB was assembled in an Ar-filled glove box (with H_2_O and O_2_ concentration below 1.0 ppm), using MWCNT/Ru cathode, along with 30 µL of a 1.2 M solution of lithium bis (trifluoromethanesulfonyl)imide (LiTFSI) in tetraethylene glycol dimethyl ether (TEGDME), LAGTP solid-state electrolyte, and a Li metal anode (0.5 mm thickness, 11 mm diameter, 99.9% purity). Following the assembly, the coin cell was placed in a testing zig designed to facilitate CO_2_ gas circulation and assess its electrochemical properties. CO_2_ gas (99.9% purity) was used for electrochemical testing. The testing zig was flushed with CO_2_ gas and linked to a battery-testing apparatus overnight to ensure LCB stabilization.

### 2.4. Characterization of Electrochemical Performance

Electrochemical performance tests were conducted using a battery test system (WBCS 3000S, WonATech, Seoul, Republic of Korea) under ambient conditions via galvanostatic discharge/charge tests. Furthermore, a cycle test was performed at a constant current density of 100 mA g^−1^ with a defined cut-off capacity of 500 mAh g^−1^. The ionic conductivity of the LATP electrolyte was determined using an EIS tester (Zive SP1, WonATech) with 150 nm thick Au electrodes affixed to both sides of the electrolyte pellets as blocking electrodes. EIS measurements were conducted at an amplitude of 50 mV across a frequency range of 0.1 Hz–1 MHz, maintaining the test environment at room temperature.

### 2.5. Material Characterizations

Raman spectroscopy (InVia Qontor, RENISHAW Ltd., Wotton-under-Edge, United Kingdom) and X-ray diffraction (XRD, D8 ADVANCE, Bruker, Billerica, MA, USA) were used for the structural characterization of LAGTP and MWCNT-Ru. Morphological and nanostructural assessments were performed using scanning transmission electron microscopy (STEM, Tecnai G2 F20, FEI, Waltham, MA, USA) and field-emission scanning electron microscopy (FE-SEM, SU-70, HITACHI, Tokyo, Japan). Energy-dispersive X-ray spectroscopy (EDS, Tecnai G2 F20, FEI) was employed to characterize the elemental composition and distribution of the MWCNT/Ru composite and LAGTP. The surface area of the MWCNTs was determined from nitrogen adsorption–desorption isotherms using the Brunauer–Emmett–Teller (BET) method (BELSORP mini X, Microtracbel, Osaka, Japan). X-ray photoelectron spectroscopy (XPS, Nexsa XPS System, Thermo Scientific, Waltham, MA, USA), FE-SEM, EDS, and STEM were used to analyze the cathodes.

## 3. Results and Discussion

**Figure 1:**
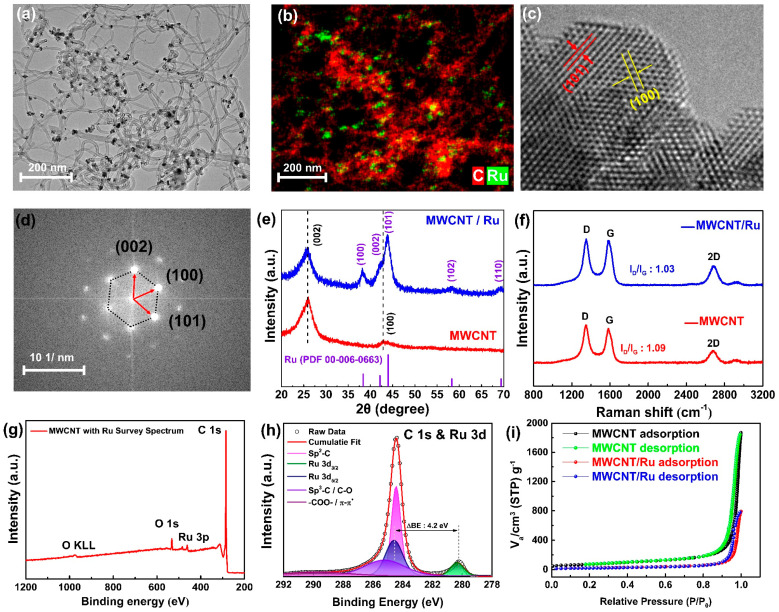
(**a**) TEM and (**b**) EDS mapping images of MWCNT/Ru. (**c**) Magnified lattice fringe image of Ru nanoparticles. (**d**) FFT pattern showing crystallographic planes of Ru. (**e**) XRD pattern of MWCNT and MWCNT/Ru. (**f**) Raman spectra of MWCNT and MWCNT/Ru. (**g**) XPS survey spectrum of MWCNT/Ru. (**h**) High-resolution C 1s and Ru 3d XPS spectra. (**i**) BET isotherm for MWCNT/Ru.

Figure 1 shows a comprehensive characterization of MWCNT-Ru in terms of its morphological, structural, chemical, and gas-adsorption properties. The STEM image in Figure 1a reveals that nanosized particles are uniformly distributed on MWCNT. EDS mapping (Figure 1b) confirmed that these nanoparticles were composed of Ru. The EDS spectrum (Figure S1) further shows the elemental composition of MWCNT/Ru, with carbon (C) at 98.68 at. % and ruthenium (Ru) at 1.32 at.%. Figure S2 shows the FE-SEM image of an MWCNT with Ru, which shows a dense MWCNT network. The crystallographic details of the Ru nanoparticles were further analyzed using STEM (Figure 1c) and fast Fourier transform (FFT) electron diffraction (Figure 1d). Lattice fringes corresponding to the (101) and (113) crystal planes of crystalline Ru, with spacings of 2.056 and 2.343 Å, respectively, are observed. From the FFT images, lattice points corresponding to the (100), (002), and (101) planes were identified [18]. Figure 1e shows the XRD patterns of MWCNT and MWCNT/Ru. The XRD pattern of the pristine MWCNTs exhibits a prominent peak at approximately 26°, corresponding to the (002) plane of hexagonal carbon. This peak is indicative of well-ordered graphitic layers typical of MWCNTs. Additionally, a less intense peak is observed at approximately 43°, attributed to the (100) plane of the hexagonal carbon structure. In the XRD pattern of MWCNT/Ru, in addition to the characteristic carbon peaks of MWCNTs, several new peaks emerge at 2θ values of approximately 38.2°, 42.2°, 43.8°, 58.3°, and 69.2°. These peaks correspond to the (100), (002), (101), (102), and (110) crystal planes of metallic Ru, respectively, in agreement with the reference pattern of hexagonal Ru (PDF 00-006-0663). The presence of these peaks confirmed the successful introduction of Ru nanoparticles on the MWCNTs. The additional Ru peaks were consistent with the hexagonal crystalline structure of Ru, corroborating the results obtained from the TEM and FFT analyses. Figure 1f shows the Raman spectra of MWCNT and MWCNT/Ru, which were used to analyze the structural properties of these materials. Both spectra exhibit two prominent peaks corresponding to the D and G bands at approximately 1350 cm^−1^ and 1580 cm^−1^, respectively. The D band, indicative of defects and disorder in the carbon structure, arises from the breathing modes of sp^3^-hybridized carbon atoms. By contrast, the G band is associated with the E_2g_ phonon mode of sp^2^-hybridized carbon atoms, representing the graphitic structural characteristics of the carbon nanotubes [19]. Additionally, both spectra exhibit a minor peak corresponding to the 2D band at around 2700 cm^−1^. The I_D_/I_G_ ratios for the MWCNT and MWCNT/Ru samples were 1.09 and 1.03, respectively, indicating no significant differences, which suggests that the carbon network of the MWCNTs remained intact and well-preserved during the synthesis. Figure 1g shows the XPS survey spectrum of MWCNT/Ru, in which peaks corresponding to C, O, and Ru can be identified. Figure S3 presents the high-resolution C 1s spectrum of pristine MWCNT. Peaks are observed at 284.4, 285.5, 286.9, 289.2, and 291.5 eV, corresponding to sp^2^-C, sp^3^-C, C-O, -COO-, and π-π* bonds, respectively [20]. Figure 1h shows the high-resolution C 1s spectrum of MWCNT/Ru, where, in addition to the peaks exhibited by pristine MWCNT, the peaks corresponding to Ru 3d_3/2_ (284.5 eV) and Ru 3d_5/2_ (280.3 eV) are present. The spin-orbit splitting value of 4.2 eV between Ru 3d_3/2_ and Ru 3d_5/2_ and the binding energy of Ru 3d_5/2_ indicated no oxidation or other chemical-state changes, signifying the formation of pure metallic Ru [21]. The surface area, adsorption–desorption characteristics, and pore size distribution of the MWCNT and MWCNT/Ru samples were characterized via BET analysis, as shown in the nitrogen adsorption–desorption isotherm graph and pore size distribution. Figure 1i presents the nitrogen adsorption–desorption isotherms for MWCNT and MWCNT/Ru. Compared to MWCNT/Ru, MWCNT exhibits higher adsorption in the relative pressure range of 0.1 to 0.8. This can be attributed to the larger surface area and porous structure of MWCNT, which facilitates more active initial adsorption. In contrast, MWCNT/Ru shows relatively lower adsorption in this range, likely due to the presence of Ru particles. The BET surface areas of MWCNT and MWCNT/Ru are 261.81 m^2^/g and 82.2 m^2^/g, respectively. The reduction in BET surface area upon Ru addition is likely because Ru particles have a relatively low surface area and high density, partially blocking the pores of MWCNT. Despite the decrease in total BET surface area, both samples exhibit hysteresis in the isotherms, a characteristic of mesoporous materials, indicative of a typical Type IV isotherm. [22,23]. This suggests that the main adsorption–desorption characteristics are still governed by the mesoporous MWCNT structure, even with the addition of Ru. The pore size distribution is shown in Figure S4. The overall distribution pattern of MWCNT and MWCNT/Ru is similar, with pore sizes mainly distributed between 30 and 80 nm, but the pore volume of MWCNT/Ru is relatively reduced. The decrease in pore volume and size in the MWCNT/Ru sample can be attributed to Ru particles dispersing on the MWCNT surface, blocking some pore structures and thus reducing the overall pore volume and size.

Multiscale characterization confirmed the formation of nanosized crystalline Ru on the MWCNTs, which did not significantly affect the original carbon structure or gas adsorption properties of the MWCNTs. This suggests that the inherent characteristics of the MWCNTs are preserved, and the catalytic effects of Ru can be effectively utilized.

**Figure 2:**
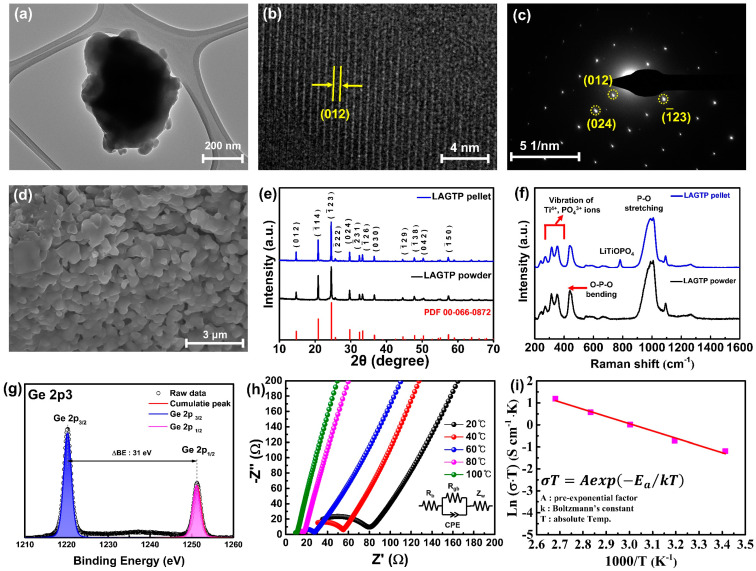
(**a**) TEM image, (**b**) magnified lattice fringe image, and (**c**) the SEAD pattern of LAGTP powder. (**d**) Magnified cross−sectional FE−SEM image of LAGTP pellets. (**e**) XRD patterns and (**f**) Raman spectra of LAGTP powder and pellets. (**g**) High−resolution XPS Ge 2p spectrum of LAGTP powder. (**h**) Nyquist plot of the LAGTP electrolyte at various temperatures, and (**i**) Arrhenius plot of the ionic conductivity of the LAGTP electrolyte.

Figure 2a shows a STEM image of LAGTP powder, which reveals the general morphology and size of the particles. The particles exhibit clear nanoscale dimensions, providing an overview of their structural arrangement. Figure S5 shows the EDS map and spectrum of LAGTP, illustrating the spatial distribution and chemical composition of its constituent elements, confirming that each element was incorporated in the intended stoichiometric amounts. The individual elemental maps for Al, Ge, Ti, P, and O show that these elements are uniformly distributed throughout the material, confirming their homogeneous integration into the LAGTP structure. Figure 2b displays a magnified image of the lattice fringes of LAGTP, with the measured d-spacing of 0.6 nm, corresponding to the (012) crystallographic plane. The distinct and sharp lattice fringes confirm the material’s high crystallinity, which indicates its well-ordered atomic structure. Figure S6 shows the FFT analysis results corresponding to Figure 2b, showing diffraction spots indexed to the (012) and (024) crystallographic planes of LAGTP. The calculated d-spacing values from the FFT analysis were 0.6 nm for the (012) plane and 0.3 nm for the (024) plane, demonstrating the well-ordered atomic arrangement of the material. Additionally, Figure 2c shows the selected area electron diffraction (SAED) pattern of LAGTP powder, where diffraction spots corresponding to the (012), (024), and (−123) planes can be observed.

The morphology of the LAGTP solid-electrolyte pellets was examined using FE-SEM. Figure S7 shows an overall image and a magnified view of the cross-section of the LAGTP pellets. The analysis showed that the pellets were dense at the microscale, with no visible pores or cracks. Figure 2d presents the cross-section of the LAGTP pellet after sintering. A comparison of the cross-sectional images of the LAGTP pellet before and after sintering is provided in Figure S8. In Figure S8a, the cross-section before sintering shows the powder compacted by mechanical pressure. In contrast, Figure S8b illustrates the grain growth and formation of an interconnected structure after sintering, providing continuous pathways for Li ions.

XRD analysis was conducted to examine the structural properties of the LAGTP powder synthesized using a solution-based method. Figure 2e shows the diffraction patterns of the LAGTP powder and pellets in the 2θ range of 10–70°, compared with LATP reference data (PDF 00-066-0872). The prominent peaks are observed at 20.9°, 24.5°, and 29.7°, corresponding to the (104), (−123), and (024) planes of LATP powder and pellets, respectively. The XRD patterns of the LAGTP powder and pellets are well consistent with the NASICON structure standard of LATP, which is also consistent with the TEM and SEAD results, confirming that LAGTP adopted a NASICON-type crystal structure. For a more detailed analysis of the structural characteristics of the LAGTP powder and pellets, the investigation focused on the 20–30° range, where the dominant peaks were located. Figure S9 shows this range’s XRD patterns of LAGTP powders and pellets. Interestingly, several weak peaks that were absent in the XRD pattern of LAGTP powder were observed at 25.9°, 27.0°, 27.4°, and 27.9°. These peaks were identified as the secondary phases of GeO_2_ and LiTiOPO_4_, which were formed during the high-temperature sintering of the LAGTP pellet [24,25]. Despite the presence of these secondary phases, their minimal intensity in the XRD pattern suggests that their formation does not significantly affect the overall performance of the pellets.

Figure 2f illustrates the Raman spectra of LAGTP powder and the sintered pellet. When comparing the spectra of the LAGTP powder and the pellet, it is evident that apart from the peak at 782 cm^−1^, which corresponds to LiTiOPO_4_, the two samples exhibit similar patterns. The emergence of the LiTiOPO_4_ peak after high-temperature sintering aligns with the previously discussed XRD results. Peaks observed at 241, 271, 315, and 355 cm^−1^ are associated with external vibrational modes, specifically the translational and librational motions of Ti^4+^ and PO_4_^3−^ ions. The peak at 439 cm^−1^ corresponds to the O-P-O bending vibration, while the internal vibrational modes observed at 969, 991, 1008, and 1093 cm^−1^ indicate the stretching vibrations within the PO_4_ tetrahedral structure. These vibrational modes are primarily observed in LATP materials, and the Raman and XRD results confirm that, apart from the subtle formation of secondary phases, there are no significant structural changes after sintering.

XPS was used to investigate the elemental composition and chemical states of LAGTP. Figure S10 shows the XPS survey spectrum of LAGTP, where peaks corresponding to Li, Al, P, Ti, O, and Ge are observed at 55.4, 74.5, 133.2, 459.6, 531, and 1220.3 eV, respectively, confirming the presence of all the constituent elements of LAGTP. Figure 2g presents the high-resolution Ge 2p spectrum of LAGTP powder, which shows distinct orbital splitting at 1220.3 eV for Ge 2p3/2 and 1251.3 eV for Ge 2p1/2. The symmetric binding energy values of Ge 2p3/2 and Ge 2p1/2 and the energy difference of 31 eV (ΔBE) between these two peaks correspond to the expected spin-orbit coupling for Ge, confirming that Ge is in the Ge4+ oxidation state [26,27]. This indicates that Ge was not reduced to a lower-valence state and was adequately integrated into the LATP crystal structure.

The electrochemical properties of the LAGTP solid electrolyte were evaluated across a temperature range of 20–100 °C using EIS. Figure 2h shows the Nyquist plots of the LAGTP electrolyte at different temperatures and the corresponding equivalent circuits. Typically, Nyquist plots of ionically conductive solid electrolytes are modeled using an equivalent circuit consisting of the grain bulk resistance (*R*_b_) and grain boundary resistance (*R*_gb_) in series and parallel combinations, along with a constant phase element (CPE) and Warburg impedance (Zw). The ionic conductivity of the LAGTP electrolyte was calculated using Equation (2).

(2) $$\sigma =\frac{1}{R}\times \frac{t}{A}$$

where σ represents the ionic conductivity of the electrolyte, *R* (*R*_b_ + *R*_gb_) is the total resistance, *t* is the thickness of the pellets, and *A* refers to the area of the blocking electrode. The total resistance and ionic conductivity of the LAGTP electrolyte at various temperatures are listed in Table 1.

Additionally, the activation energy was derived from temperature-dependent EIS measurements using the Arrhenius Equation (3):

(3) $$\sigma T=Aexp\langle -\frac{E_{a}}{kT}\rangle$$

where *A*, *E*_a_, *k*, and *T* denote the pre-exponential factor, activation energy, Boltzmann constant, and absolute temperature, respectively. From the slope of the Arrhenius plot in the Figure 2i, the activation energy for ion conduction in LAGTP was calculated to be approximately 0.25 eV, which is notably lower than the activation energy typically reported for LATP [28,29,30]. This reduction in activation energy suggests that Ge doping improves the ionic mobility in LAGTP. The ionic radius of Ge^4+^ (0.53 Å) is smaller than that of Ti^4+^ (0.605 Å). When Ge partially replaces Ti^4+^ in the LATP structure, the smaller Ge^4+^ ions induce a structural distortion. This structural adjustment helps create interconnected, low-energy pathways, facilitating Li-ion transport via a percolation network [31]. Introducing local distortions generates a distribution of site energies, enabling overlapping energy levels between neighboring sites. This overlap creates a percolation pathway, enabling Li ions to migrate more freely through the electrolyte and enhancing diffusion without requiring a significant increase in cell size.

**Figure 3:**
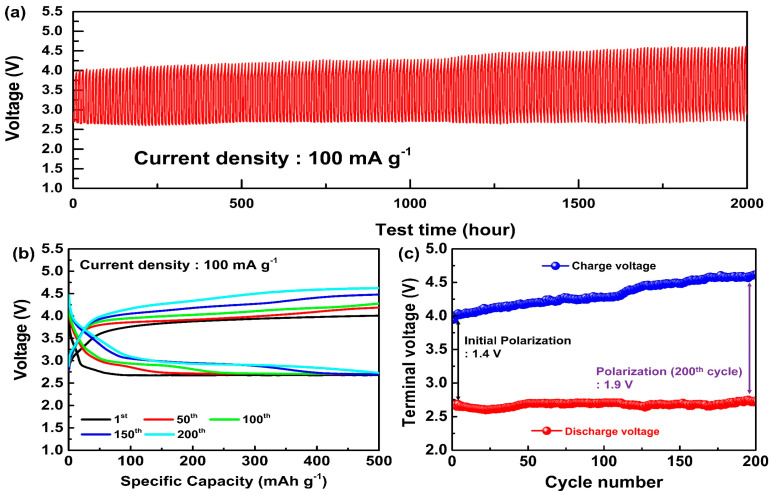
(**a**) Voltage−time, (**b**) voltage−specific capacity, and (**c**) terminal voltage-cycle number graph of LAGTP applied LCBs at a current density of 100 mA/g.

A schematic illustration of the Li-CO_2_ battery system is presented in Figure S11, highlighting the LAGTP solid electrolyte and the MWCNT/Ru cathode, along with the reaction mechanisms of CO_2_ reduction during discharge and Li_2_CO_3_ decomposition during charge. This configuration was used in the electrochemical tests to assess the battery’s performance. Cycling tests were conducted to evaluate the electrochemical performance of the LCB based on the LAGTP solid electrolyte and Ru-enhanced MWCNT cathode. The current density and specific capacity were calculated using the mass of the MWCNT/Ru cathode. The cycling tests were performed at a current density of 100 mA/g and a capacity limit of 500 mAh/g. Figure 3a shows the overall cycling profile of the LCB over 200 cycles, demonstrating its successful operation over 2000 h of testing. The battery components underwent minimal degradation during the cycling test. Figure 3b shows the galvanostatic charge–discharge profiles over various cycles (1st, 50th, 100th, 150th, and 200th cycles). As the cycles progressed, changes in the voltage plateaus for both discharging and charging were observed. Figure 3c illustrates the change in terminal voltage during cycling. Initially, the discharge voltage was approximately 2.6 V. The charge voltage was approximately 4.0 V, resulting in a polarization of approximately 1.4 V. After 200 cycles, the discharge voltage increased to 2.7 V, and the charge voltage increased to 4.6 V. Additionally, the polarization increased to 1.9 V, marking a 0.5 V increase from that at the initial cycle. While the discharge terminal voltage remains relatively stable, the charge terminal voltage steadily increased from 4.0 V to approximately 4.6 V after 200 cycles. The initial terminal voltage (4.0 V) was significantly lower than the typical charge-terminal voltage (4.5 V) observed in conventional MWCNT-based LCBs, which can be attributed to the catalytic activity of Ru [32,33]. NASICON-type solid electrolytes tend to degrade faster when high voltages are sustained [34]; therefore, the formation of a lower charge–voltage plateau due to the introduction of Ru contributes to the stability of the solid electrolyte in the LCB. Table 2 presents the performances of LCBs reported to date that use solid electrolytes. The LCB utilizing LAGTP and MWCNT/Ru exhibited excellent cycling performance. This improvement is attributed to the enhanced stability of the solid electrolyte, which was achieved through Ge doping [35], and the catalytic effects of Ru, enabling operation within a lower voltage range, leading to the development of a high-performance LCB.

**Figure 4:**
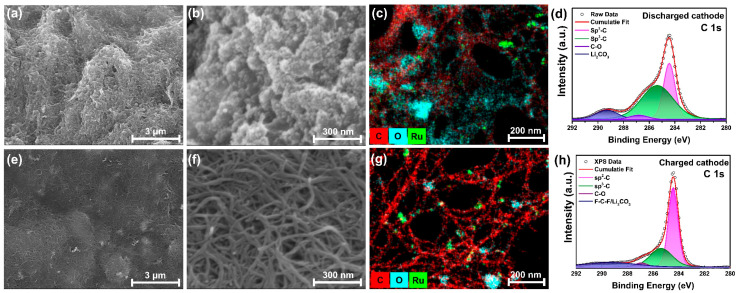
FE-SEM images of (**a**,**b**) the discharged cathode and (**e**,**f**) the charged cathode, showing the surface morphology. EDS mapping of (**c**) the discharged and (**g**) charged cathodes. XPS spectra of the C 1s peak for (**d**) the discharged and (**h**) charged cathodes.

A post-cycle analysis was conducted to validate the performance of the LCB. Figure 4a,b show FE-SEM images of the cathode after discharge. In Figure 4a, discharge products can be observed to form on the MWCNT matrix. Figure 4b provides a higher-magnification view, where granular discharge products covering the surface are visible. Figure 4e,f show the FE-SEM images of the cathode after charging. According to Equation (1), the discharge products decompose after charging, and the surface returns to that of pristine MWCNT/Ru, as shown in Figure S2. Figure 4c,g present the EDS mapping images of the cathode after discharging and charging, respectively. The mapping results illustrate the spatial distribution of C, O, and Ru across the cathode surface. C and Ru from MWCNT/Ru were observed in the discharged and charged states. In Figure 4c, which represents the discharged state, O from the discharge product Li_2_CO_3_ is dominant. Conversely, as shown in Figure 4g, the O concentration decreases significantly after charging, indicating the decomposition of the discharge products. However, the localized areas show the presence of irreversibly formed Li_2_CO_3_. As the cycling progressed, the accumulation of Li_2_CO_3_ contributed to increased polarization. XPS analysis was performed to verify the chemical states of the cathode and the discharge products after charge and discharge tests. Figure S12 presents high-resolution XPS spectra of Li 1s and Ru 3p in the cathode after charge and discharge. Figure S12a shows the Li 1s high-resolution XPS spectrum for the charged and discharged states of the cathode. In the discharged state, peaks at Li_2_CO_3_ at 54.5 eV corresponding to Li_2_CO_3_ and a trace peaks at 55.6 eV for LiTFSI are observed. The presence of LiTFSI is attributed to residual lithium salt remaining after the cleaning process. After charging, the Li 1s spectrum reveals only a minor peak for LiTFSI, indicating that the discharge product, Li_2_CO_3_, has decomposed [41]. Figure S12b displays the Ru 3p high-resolution XPS spectrum for both charged and discharged states, with identical peak positions observed in both cases. The peaks are located at Ru^0^ 3p_3/2_ (462.0 eV), Ru^4+^ 3p_3/2_ (465.0 eV), Ru^0^ 3p_1/2_ (484.5 eV), and Ru^4+^ 3p_1/2_ (487.6 eV) [42]. Figure 4d,h show the high-resolution C 1s XPS spectra of the discharged and charged cathodes, respectively. In the discharged cathode, the C 1s spectrum shows peaks corresponding to sp^2^–C (284.4 eV), sp^3^–C (285.4 eV), and C–O (286.8 eV) bonds and Li_2_CO_3_ (289.3 eV), indicating the formation of lithium carbonate during discharge [43,44]. After charging, the XPS spectrum revealed a change in the chemical environment, with peaks corresponding to MWCNT and F-C-F from the PVDF binder [45], whereas the Li_2_CO_3_ peak diminished but remained. This indicates that, although Li_2_CO_3_ decomposed during the charging process, some irreversible Li_2_CO_3_ persisted. These characterization results confirm the occurrence of reversible reactions, as shown in Equation (1), while also indicating the presence of irreversible Li_2_CO_3_, which contributes to the increase in polarization during cycling.

## 4. Conclusions

In this study, we demonstrated the successful integration of Ge-doped LAGTP solid electrolytes and MWCNT/Ru cathodes in LCBs. Ge doping in LAGTP resulted in significantly higher ionic conductivity, achieving a value of 1.04 × 10^−3^ S/cm at 25 °C by lowering the activation energy and expanding the Li-ion migration channels while maintaining the NASICON-type structure. Ru nanoparticles anchored on the MWCNT enhanced the catalytic activity for Li_2_CO_3_ decomposition, reducing the overpotential and extending the cycling performance of the LCB. Electrochemical tests revealed that the LCB assembled with LAGTP and MWCNT/Ru maintained stable operation over 200 cycles, highlighting the combined effects of enhanced electrolyte stability and improved cathode performance. Furthermore, post-cycle analysis confirmed the reversible electrochemical reaction of CO_2_ with Li, although a minor accumulation of irreversible Li_2_CO_3_ contributed to a gradual increase in polarization. This study demonstrates the potential of using Ge-doped NASICON-type electrolytes and Ru catalysts to enhance the electrochemical stability and performance of LCBs, offering a promising strategy for future energy storage technologies contributing to CO_2_ sequestration.

## Figures and Tables

**Table 1:** Total resistance and total ionic conductivity of LAGTP at various test temperatures.

Temperature (°C)	Total Resistance (Ω)	Total Ionic Conductivity (mS cm^−1^)
20	79.9	1.04
40	53.2	1.56
60	27.3	3.03
80	16.5	5.02
100	9.4	8.80

**Table 2:** Cycle performance of Li-CO_2_ batteries with inorganic solid electrolytes at room temperature.

S.No	Cathode Material	Inorganic Electrolyte	Cycle Life	Ref.
1	MWCNT	LATP	50	[36]
2	SWCNT/RuO_2_	LAGP	30	[37]
3	Ru/CNT	LAGP	45	[33]
4	MWCNT	LAGTP	60	[38]
5	MWCNT /Ru	LATP	50	[32]
6	Co_3_O_4_-derived MOF	LATP	100	[39]
7	Fe_3_C/N-doped CNT	Zn-doped LATP	180	[40]
8	MWCNT/Ru	LAGTP	200	This work

## Data Availability

The original contributions presented in the study are included in the article/Supplementary Materials; further inquiries can be directed to the corresponding authors.

## Supplementary Materials

The following supporting information can be downloaded from: https://www.mdpi.com/article/10.3390/nano14231894/s1, Figure S1. EDS spectrum and elemental composition of MWCNT/Ru; Figure S2. FESEM images of the MWCNT with Ru; Figure S3. High-resolution C 1s spectrum of pristine MWCNT; Figure S4. Pore size distribution of MWCNT and MWCNT/Ru; Figure S5. Energy-dispersive X-ray spectroscopy (EDS) elemental mapping of LAGTP powders, indicating the distribution of (a) all detected elements (Al, Ge, P, Ti, and O), (b) Al, (c) Ge, (d) O, (e) P, and (f) Ti. (g) EDS spectrum of LAGTP and chemical composition; Figure S6. FFT image of the LASTP powder corresponding to Figure 2b; Figure S7. (a) Cross-sectional FESEM image of the LAGTP pellet attached to the carbon tape (a) and (b) magnified FESEM image of the pellet; Figure S8. Cross-sectional FESEM image of an LAGTP pellet (a) before and (b) after sintering; Figure S9. Magnified XRD spectra of LAGTP powder and pellets in the 20–30°; Figure S10. Survey spectrum of the LAGTP powder. Figure S11. Schematic illustration of the Li-CO2 battery and its electrochemical reactions; Figure S12. High-resolution XPS spectrum of (a) Li 1s and (b) Ru 3p in the cathode after charge and discharge.
