# Supplementary material for: Enhancing the Performance and Stability of Li-CO2 Batteries Through LAGTP Solid Electrolyte and MWCNT/Ru Cathode Integration
Source: Nanomaterials (Basel). 2024 Nov 26;14(23):1894. doi: 10.3390/nano14231894 (PMC11643571; doi:10.3390/nano14231894)
Supplement: Supplementary file 1 [file nanomaterials-14-01894-s001.zip › nanomaterials-3299245-supplementary.pdf]

# Supplementary Materials

# Enhancing the Performance and Stability of Li-$CO_2$ Batteries Through LAGTP Solid Electrolyte and MWCNT/Ru Cathode Integration

Dan Na [1], Dohyeon Yu [1], Hwan Kim [1], Baeksang Yoon [1], David D. Lee [2] and Inseok Seo [1,*]

[1] *Department of Electronic and Information Materials Engineering, Division of Advanced Materials Engineering, Research Center of Advanced Materials Development, Jeonbuk National University, Jeonju 54896,* Republic of Korea

[2] *Aerospace Engineering, Iowa State University, Ames, IA 50011, USA*

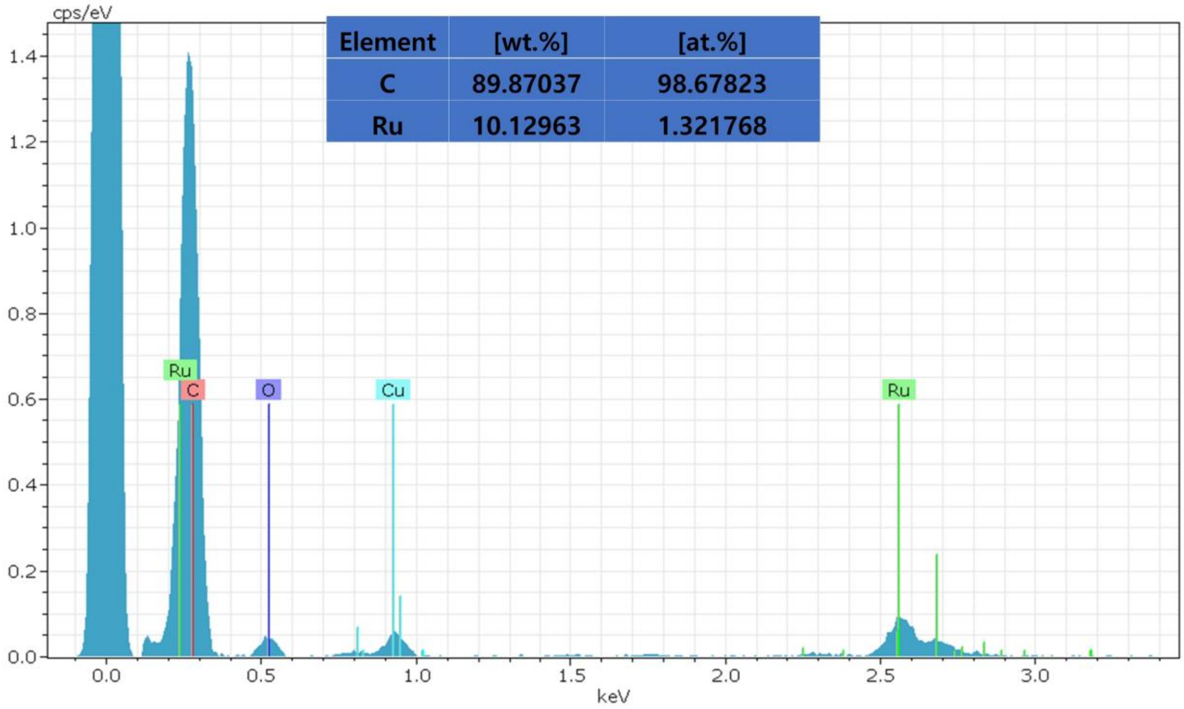


**Figure S1.** EDS spectrum and elemental composition of MWCNT/Ru.

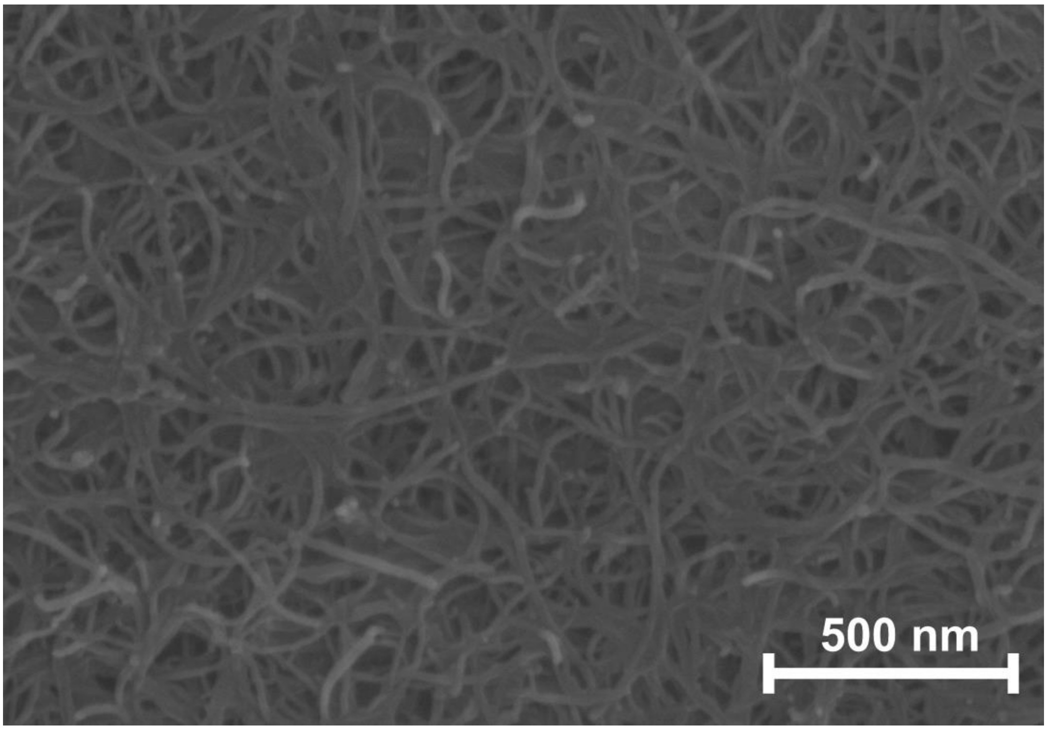


**Figure S2.** FE-SEM images of an MWCNT with Ru.

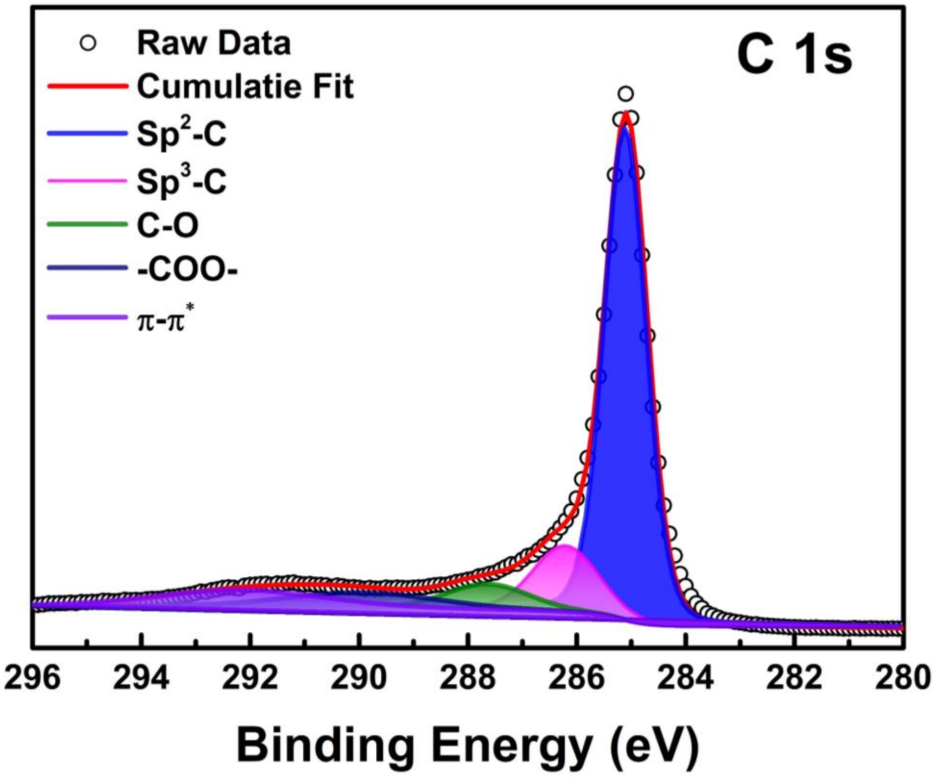


**Figure S3.** High-resolution C 1s spectrum of pristine MWCNT.

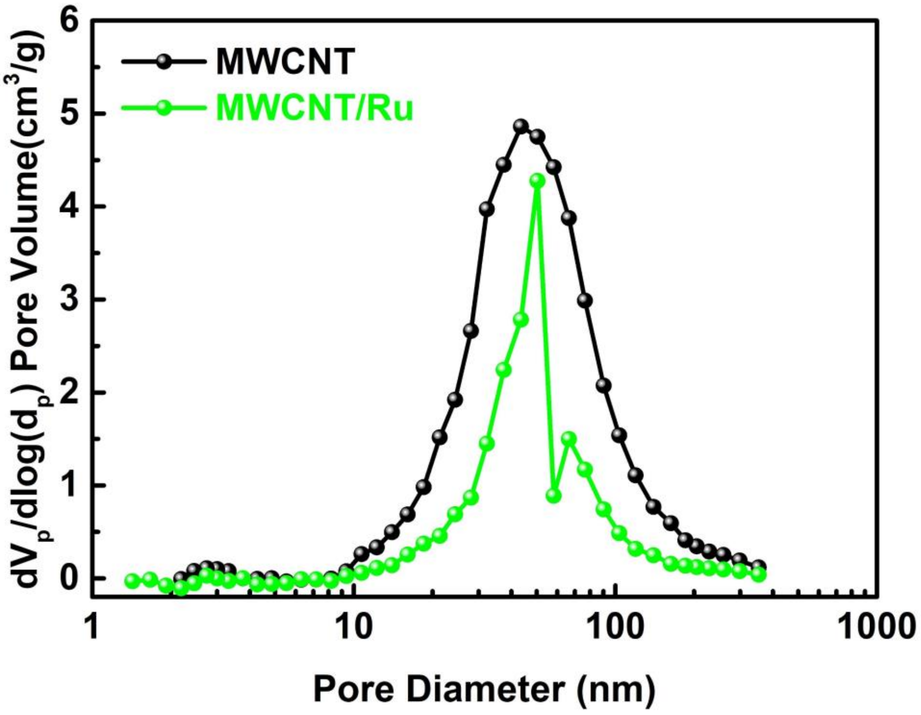


**Figure S4.** Pore size distribution of MWCNT and MWCNT/Ru.

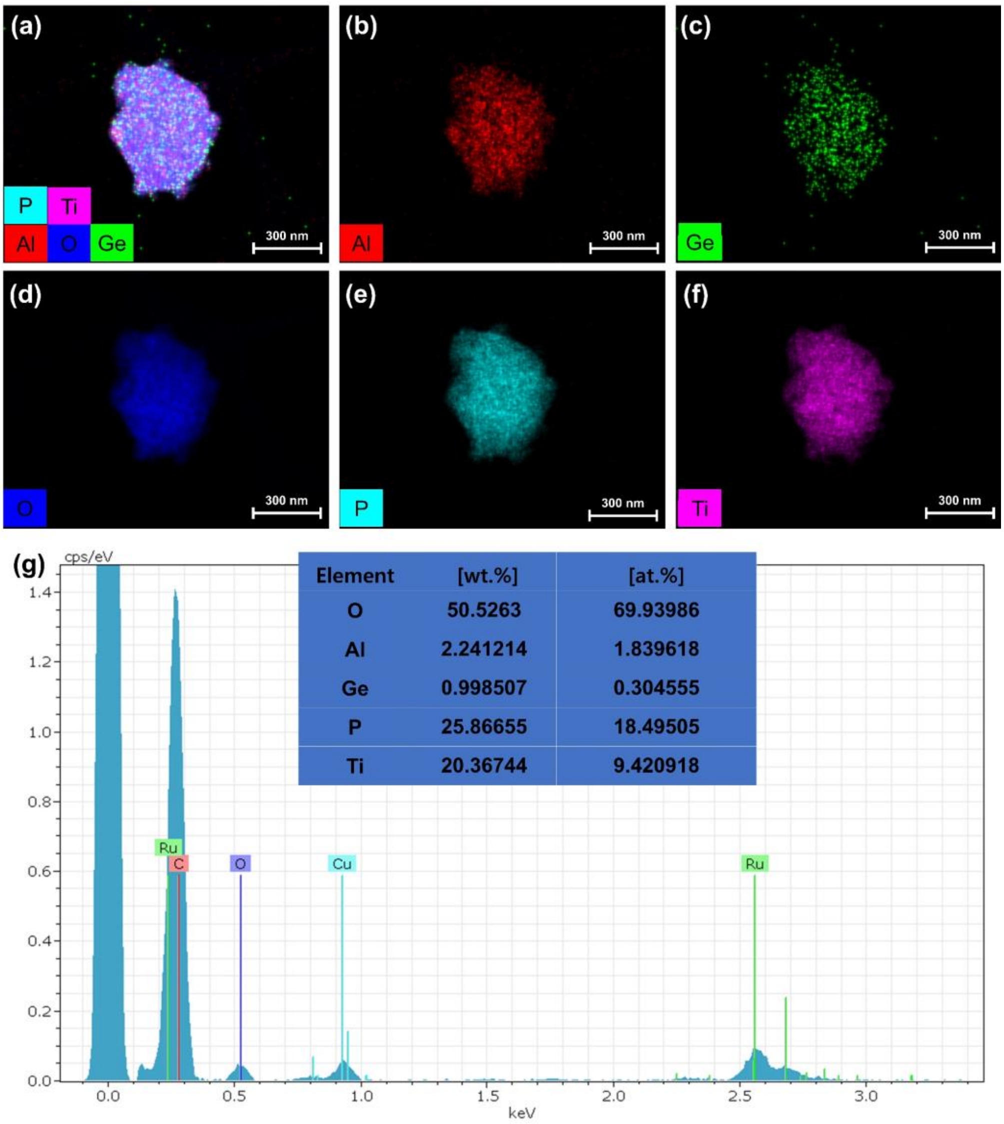


**Figure S5.** Energy-dispersive X-ray spectroscopy (EDS) elemental mapping of LAGTP powders, indicating the distribution of (a) all detected elements (Al, Ge, P, Ti, and O), (b) Al, (c) Ge, (d) O, (e) P, and (f) Ti. (g) EDS spectrum of LAGTP and chemical composition.

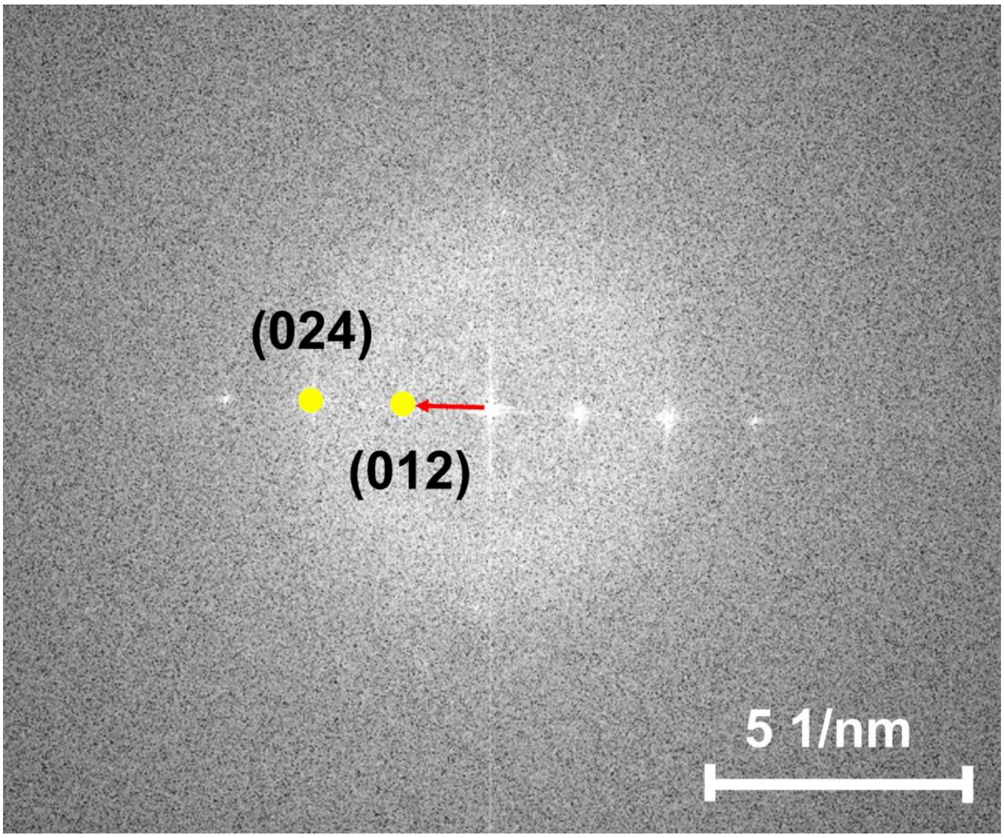


**Figure S6.** FFT image of LASTP powder corresponding to Figure 2 (b).

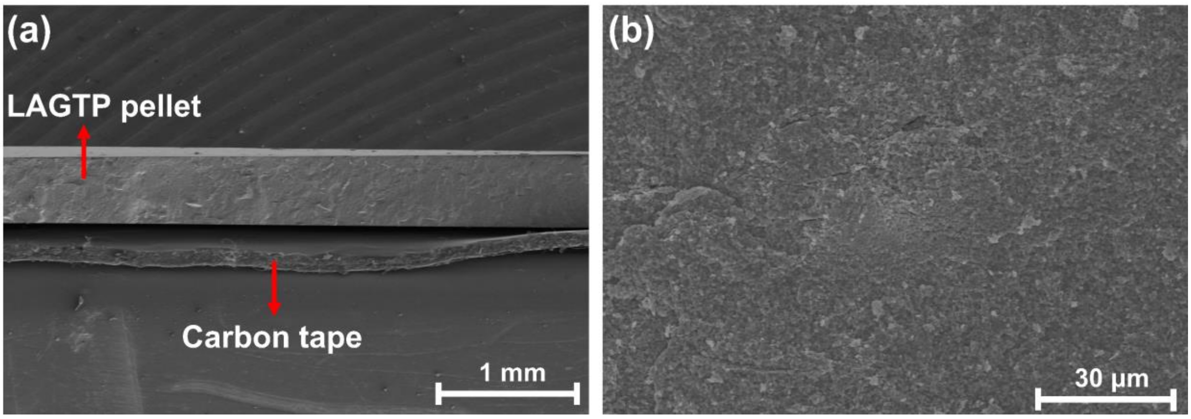


**Figure S7.** Cross-sectional FESEM image of the LAGTP pellet attached to the carbon tape (a) and (b) magnified FESEM image of the pellet.

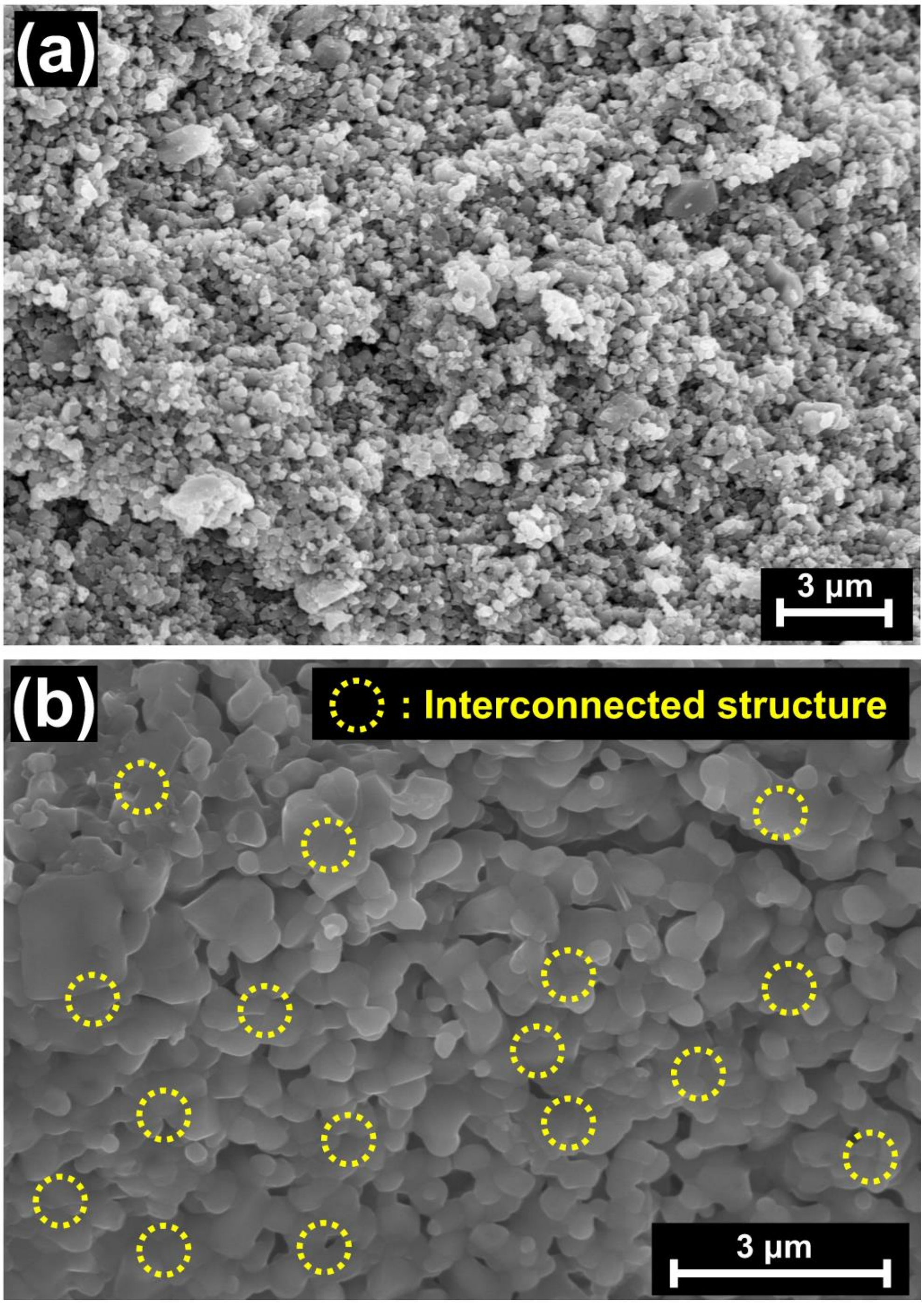


**Figure S8.** Cross-sectional FESEM image of an LAGTP pellet (a) before and (b) after sintering.

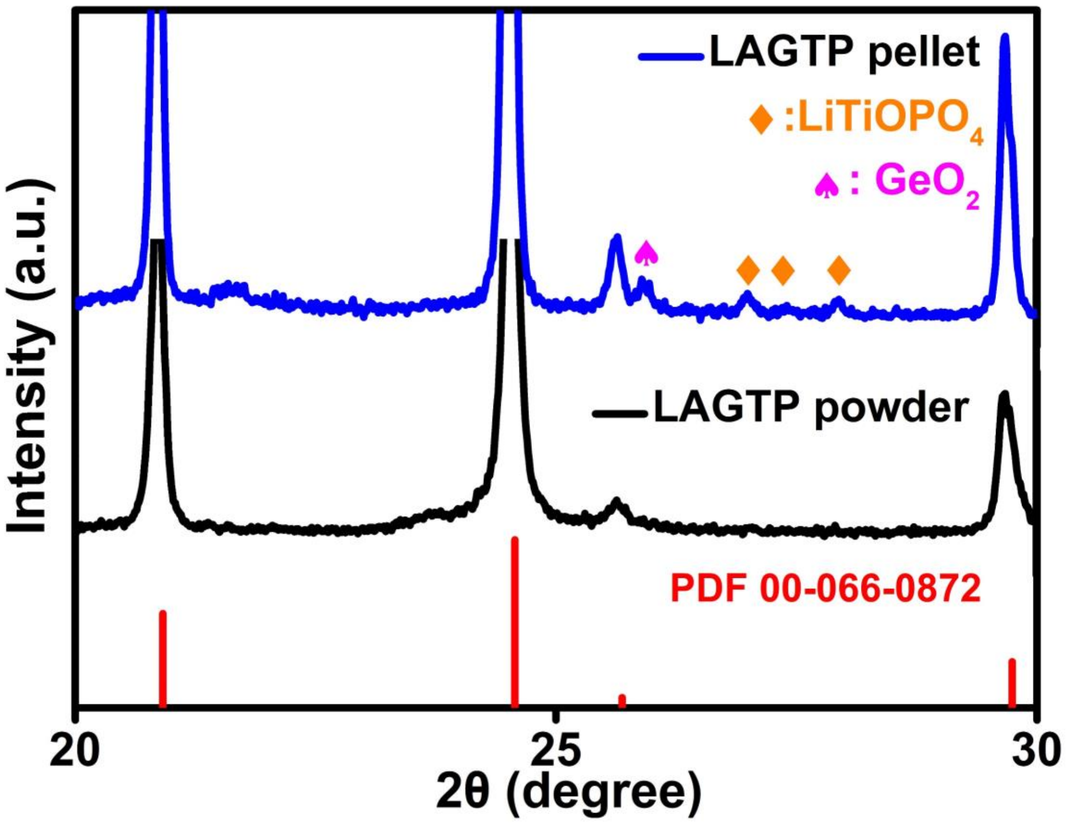


**Figure S9.** Magnified XRD spectra of LAGTP powder and pellets in the range of 20–30°.

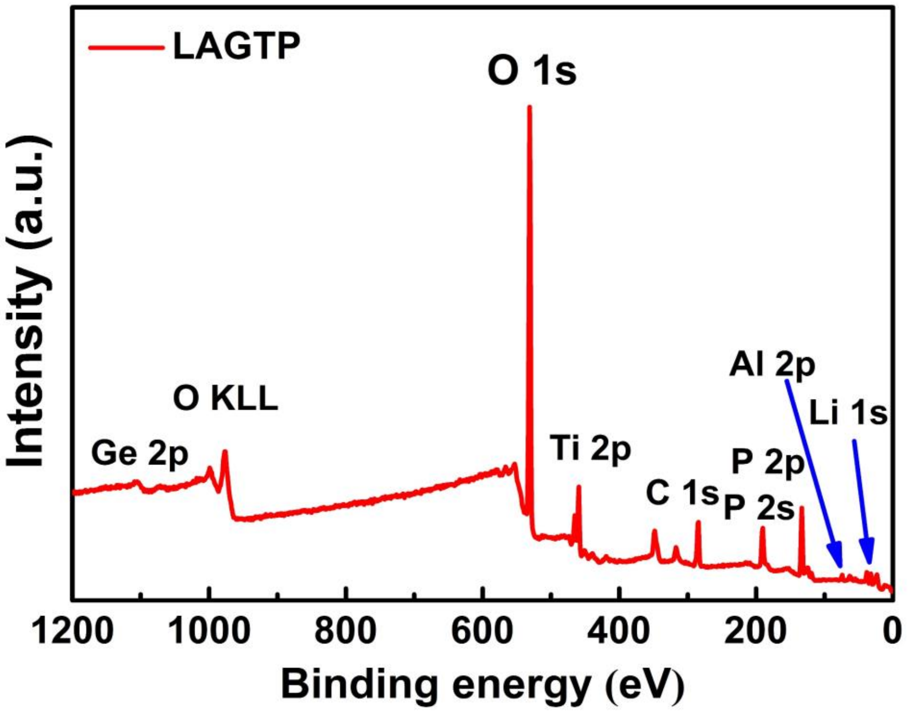


**Figure S10.** Survey spectrum of the LAGTP powder.

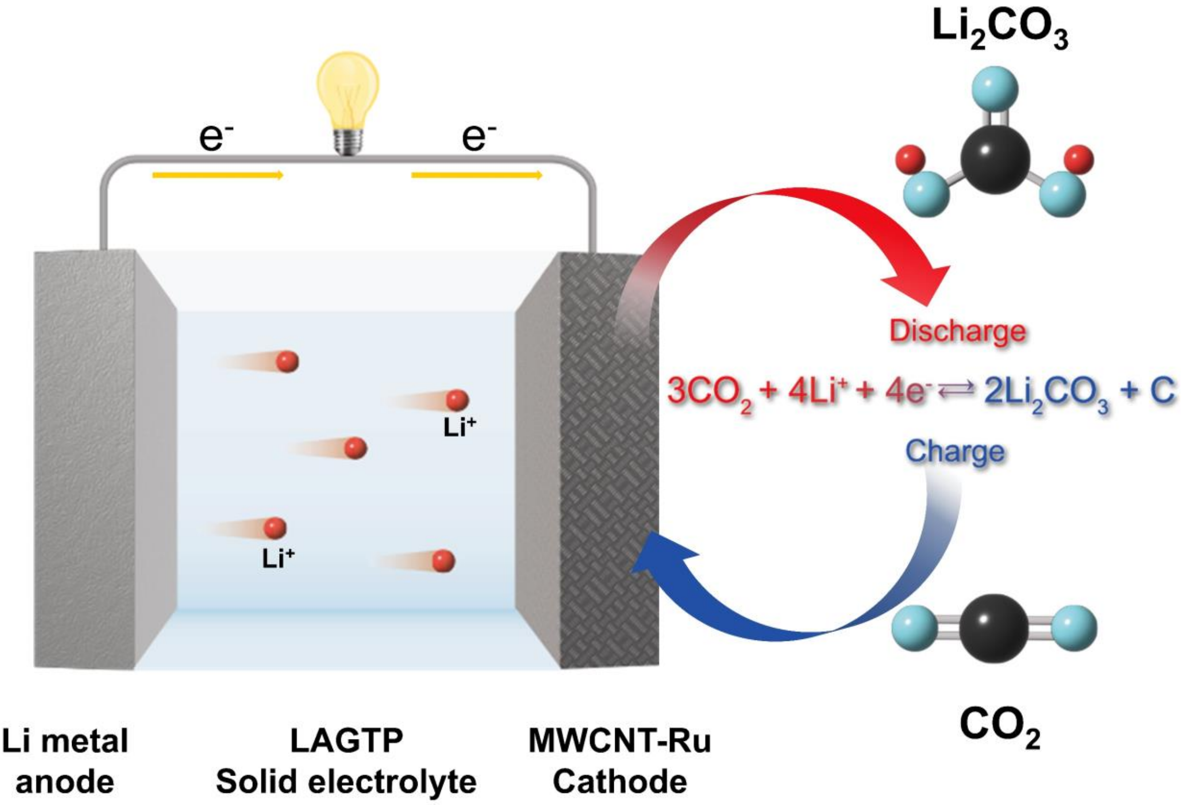


**Figure S11.** Schematic illustration of the Li-$CO_2$ battery and its electrochemical reactions.

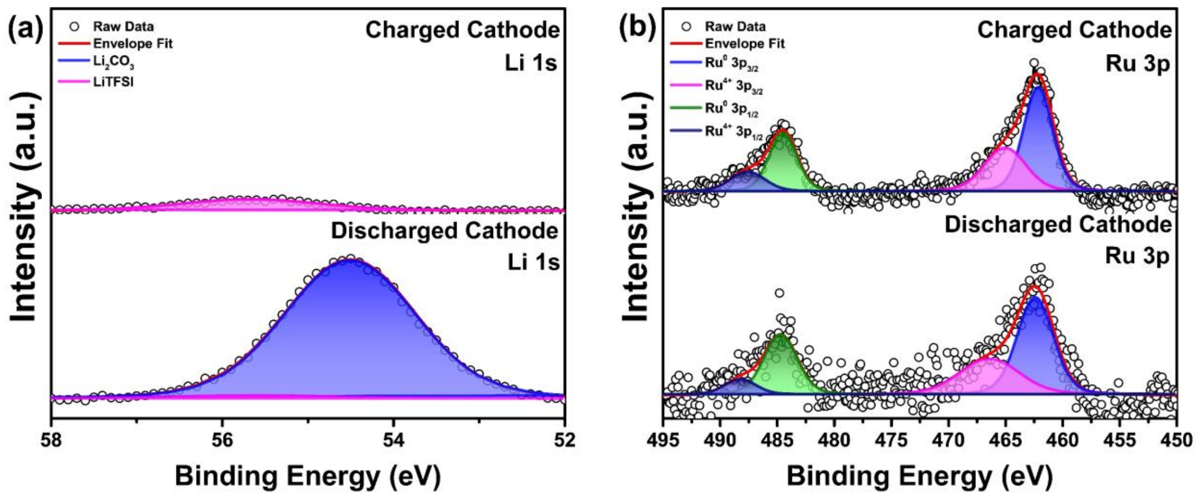


**Figure S12.** High-resolution XPS spectrum of (a) Li 1s and (b) Ru 3p in the cathode after charge and discharge.
